# The future of cancer research after COVID-19 pandemic: recession?

**DOI:** 10.2217/fon-2020-0397

**Published:** 2020-05-29

**Authors:** Hampig Raphael Kourie, Roland Eid, Fady Haddad, Marwan Ghosn, Dolla Karam Sarkis

**Affiliations:** 1 Hematology-Oncology Department, Faculty of Medicine, Saint Joseph University of Beirut, Beirut, Lebanon; 2 Microbiology Department, Faculty of Pharmacy, Saint Joseph University of Beirut

**Keywords:** cancer, COVID-19, ethics, funds, research, SARS-CoV-2, trials

By the end of 2019, cancer research was at one of its highest productivity and efficiency peaks with a tsunami of targeted therapies and approved immunotherapies in different malignancies. Numerous drugs in pharmaceutical companies’ pipeline were being evaluated in thousands of clinical trials around the world. With the outbreak of SARS-CoV-2 infection in China in December 2019 which quickly grew into a pandemic, the majority of countries across Asia, Europe and America have been affected on medical, social and economic levels. During this period, the main challenge for the healthcare system in these countries was to limit the spread of the virus among people, provide the best care to infected patients and decrease the number of possible deaths. Suddenly, we saw a shift in priority levels where the fight against SARS-CoV-2 infection has become more urgent and crucial than the fight against cancer. As a result, cancer research has slowed considerably.

In fact, during the COVID-19 pandemic, strict public health measures, including a total lockdown, were put in place by national authorities in different countries to stop the spread of the virus. The quarantine measures also affected cancer researchers, as they started to work in shifts and to lack run out of supplies. Thus, the researchers were confronted with the need to make decisions on the continuation or interruption of the trials [1].

Moreover, cancer centers have adopted a new system for selecting clinical trials. They prioritized clinical trials based on their significant evidence of clinical activity and withheld early phase clinical trials, namely Phase I trials where the clinical benefit is really dismal. Many cancer centers have decided to freeze the activation of new trials and have stopped inpatient studies. Different pharmaceutical companies have decided to delay the start of new clinical trials, suspended enrollment in some other trials or postponed the activation of new sites in trials continuing enrollment. Meanwhile, the US FDA has asked trials’ sponsors to be more flexible about protocols. The FDA has authorized remote communication with patients for follow-up and to make changes and exceptions to trials’ protocols in order to reduce multiple hospital or routine follow-up visits. Other measures have also been taken, such as switching from inpatient to outpatient treatment, postponing or skipping doses, and skipping routine blood draws and biopsies in clinical trials [2]. Clear amendments should be added to research protocols of ongoing trials, during a well-defined period of time related to COVID-19 pandemic according to each region, to decrease the possible criticism for exceptions and delays in future published results.

In addition, some cancer research laboratories have chosen to shift their experiments with antitumor agents, from the treatment of cancer to the exploration of their therapeutic benefits in SARS-CoV-2 infection. For example, Chinese trials are evaluating the role of the antiVEGF agent bevacizumab, the multiple myeloma drug thalidomide, the PD-1 inhibitor camrelizumab and other antitumor drugs in the treatment of SARS-CoV-2 infections. Other scientists working in the field of biomarkers in oncology are now rotating their research to develop better techniques for diagnosing COVID-19 infections [3]. It is important to assure a fast reshifting of these scientists and laboratories to oncology research after controlling the SARS-CoV-2 outbreak and discovering a potential vaccine.

Respect for the ethics of clinical research remains mandatory in the management of clinical trials during this pandemic. Three critical considerations in cancer research were highlighted by Shuman *et al.* in the *Oncologist*. The first is the nonabandonment of patients on experimental protocols of oncology with curative or palliative intent, mainly those who have a strong interest in remaining in the trial. The second is making an effort to decrease the risk of infection by COVID-19 (flattening of the curve) by avoiding unnecessary exposures related to interventions, assessments and visits to hospital and clinics. Finally, psychosocial support for the patient and the research team is of the utmost importance in the midst of this global epidemic [4].

It is worth mentioning the exponentially increasing number of articles published since February 2020 related to cancer and SARS-CoV-2 pandemic. The vast majority of these papers are related to the strategies adopted for risk reduction and the management of cancer patients during this viral outbreak, as well as the characteristics of cancer patients (epidemiological, clinical and radiographic) and their influence on outcomes [5,6]. The priority of oncology researchers during this pandemic has become focused on reducing the risk of infection by COVID-19 among cancer patients, rather than finding new therapies for them.

Cancer research will be facing one of the most critical recession in its history due to the SARS-CoV-2 outbreak. The consequences will be crucial not only on the short-term (next few months) but also on the long-term (following years). From an economical perspective, medical research can be funded by the government, cancer societies, pharmaceutical companies or other charitable entities, such as in the UK. During this pandemic, governments in Europe, UK, USA and Australia, maintained their funding for biomedical research, prolonged deadlines for grants submissions and virtualized peer-review panels. However, research that is driven by charitably funded entities or public fundraising, was subject to budget restriction following the decline in economic growth imposed by the COVID-19 outbreak. In fact, Cancer Research UK, the charity responsible for funding about half of all UK cancer research, cut its research budget by 10% this year while also postponing the funds for new research projects for at least the first half of 2020. This resulted from the reduction of supporters’ donations, on which Cancer Research UK relies [7]. The American Cancer Society also warned about the detrimental effects of the pandemic on the economy and donations, limiting its ability to normally fund grants for the next cycle of 2020. Furthermore, the Canadian Cancer Society estimates a drop in donation of around half of its budget in the upcoming year, around 100 million Canadian dollars [7].

The worldwide economic crisis secondary to SARS-CoV-2 outbreak will also impact negatively, but indirectly, cancer research on the long-term. After the resolution of the outbreak, opening of the borders between countries and resumption of daily activities, governments will be facing a big threat, that of unemployment and poverty. At this point, the priority of every government will be to guarantee the security of its citizens, by creating job opportunities, assuring a minimal income for individuals who lost their jobs due to the pandemic and providing adequate healthcare for people in need. Long-term plans are needed to restore the balance that was present before this crisis and this puts a huge financial burden on every government, which may lead to budget reduction in other domains, such as cancer research, in order to supply these plans.

Priorities in research investment are also changing, with more new funds attributed to manage the COVID-19 outbreak and to establish new strategies for preventing other potential pandemics in the future. In fact, following the spread of SARS-CoV-2, efforts of the WHO were focused on fighting this scourge. Scientific research tackling the various aspects of COVID-19 represented an urgent priority and an essential component of the WHO response to this pandemic. Therefore, funds and necessary resources have been coordinated and mobilized to initiate COVID-19-oriented research [8].

Moreover, many opportunities related to the development of new cancer drugs will be missed because of the redirection of funds for the study of SARS-CoV-2. In fact, a part of the governmental funds and resources of pharmaceutical companies that are dedicated to cancer therapies trials will be transferred to studies on COVID-19 detection, testing, treatment and vaccines. This will result in a slower evolution of cancer trials, a deferral in the expected time of study completion and therefore awaited results. In addition, the resulting changes and amendments to the protocols of ongoing trials represent a possible ground for criticism at the time of publication.

The coordination between multiple entities is required for the management of research during the crisis. First, the capacity of governments plays an essential role. For instance, the Australian government did not impose restrictions on the funds for medical research, but rather, they adapted their use by accelerating the delivery of funds related to COVID-19 research while slowing down some resources dedicated to other research activities during 2020. Similarly, in Europe, Advanced Grants call was opened with a total budget of around €500 million, a strategy aiming to prevent any negative impact on current grantees or future applicants. In the UK, research applicants benefited from an extension to deadlines on funding opportunities, which gave them additional time for the submission of their applications [7]. Second, the FDA released priorities regarding COVID-19 and cancer patients, that included statements on research management. On the level of clinical trials, the FDA recognized that amendments may be necessary such as keeping patients informed of any modifications that might affect them, assisting sponsors in assuring the safety of the individuals included in the trial, while maintaining compliance with good clinical practice. On the level of drug development, a coordination is needed between drug developers, academic investigators and patient advocates to allow proper review of drugs, biologics and devices for cancer. On the level of patient care, the FDA continued to process Expanded Access requests that allow cancer patients to access investigational therapies for the treatment of their cancer when no other alternative treatments are available and there is no opportunity for the patient to enroll in a clinical trial [9]. Third, it will be necessary to limit the negative impact of this pandemic on cancer research by encouraging pharmaceutical companies and cancer centers to increase their investment in cancer research, by facilitating loans and grants administered by governments, nonprofit organizations and cancer patient associations. Finally, distancing represents one of the inevitable obstacles imposed by the SARS-CoV-2 pandemic, almost universally. Initially, it led to a reduction or cessation of meetings, which delayed decision making in different fields, namely cancer research. Therefore, virtual meetings emerged as a possible solution. For example, the US National Institutes for Health maintained research continuity with no disruption by organizing online meetings with the different study sections, reviewing the evolution of the trials and making decisions [7].

To conclude, the load of research, publications and new trials in oncology will most probably decrease on short and long-term due to this pandemic. Cancer researchers, although stuck in the middle of this pandemic, should counter this impending recession by combining their efforts to establish a clear road map to ensure a smooth and effective revival of cancer research immediately after the lockdown ends and new recommendations for trials, that will start, should be added to the protocols concerning the period.
